# 

**Published:** 1907-11-16

**Authors:** 


### RESIDENT MEDICAL OFFICERS' DEPARTMENT.

### NOTES, QUESTIONS, AND COMMENTS.

The editor of the Resident Medical Officers' Department of The Hospital invites contributions to this column both from resident medical officers and from other members of the profession. Short articles, notes, queries, or suggestions bearing upon this branch of medical practice will always receive careful consideration, and those that are suitable will be published in due course. Correspondence, short and to the point, is particularly invited, as by this means the value of the R.M.O. Department will be greatly increased.

Articles, whether in the form of letters to the editor or otherwise, should in no case exceed 600 words in length, and should, if possible, be shorter than this. Postcards with short notes, questions, or notifications of recent hospital appointments will be welcomed.

### THE GRADUATES' MEDICAL SCHOOLS.

### Diary for the week November 18 to 23.

### MEDICAL GRADUATES' COLLEGE AND POLYCLINIC, Chenies Street, W.C.

**Daily** (except Saturday): Clinics, Demonstrations and Practical classes.

**Lectures at 5.15 p.m.**

November 18, Dr. Chalmers Watson, **A Purin-Free Diet.**

November 19, Sir Douglas Powell, **Senile Respiratory Disorders.**

November 20, Mr. Donald Armour, **Head Injuries.**

November 21, Mr. A. W. Mayo Robson, **Pancreatic Catarrh and Interstitial Pancreatitis in their relation to Catarrhal Jaundice and also to Glycosuria.**

### THE POST-GRADUATE COLLEGE, West London Hospital, Hammersmith, W.

**Daily at 2:** Medical and Surgical Clinics; **at 2.30:** Operations.

**Special Departments at 10** (Tuesday, Wednesday, Friday), and daily at **2.**

**Lectures at 5 p.m.**

November 18, Mr. Baldwin, **Practical Surgery** (Lecture V.).

November 19, Dr. Seymour Taylor, **Neuralgiæ and their Treatment.**

November 20, Mr. Lloyd Williams, **Fractures of the Jaw.**

November 21, Mr. Dunn, **Cases of Diseases of the Eye.**

November 22, Dr. Abraham, **Cases of Skin Disease.**

### LONDON SCHOOL OF CLINICAL MEDICINE, Seamen's Hospital, Greenwich.

**Medical and Surgical Clinics.**—Demonstrations and special departments daily.

**Lectures.**

November 18, **3.15 p.m.,** Mr. William Turner, **Hepatic Abscess.**

November 20, **2.15 p.m.,** Dr. F. Taylor.

### WEST END HOSPITAL FOR DISEASES OF THE NERVOUS SYSTEM, Welbeck Street, W.

**At 5 p.m.**—November 21, Mr. Laming Evans, **The Indications for Arthrodesis of the Shoulder-joint.**

### NORTH-EAST LONDON POST-GRADUATE COLLEGE, Prince of Wales's Hospital, Tottenham, N.

**Surgical, Medical and Special Clinics** daily, Saturdays excepted.

**Special Lectures and Demonstrations.**

November 19, **4.30 p.m.,** Mr. Howell Evans, **Cancer of the Face.**

November 21, **5 p.m.,** Dr. Squire, **Chest Cases** (at Mount Vernon Hospital, Hampstead, N.W.).

### NATIONAL HOSPITAL FOR THE PARALYSED AND EPILEPTIC.

**At 3.30 p.m.**

November 19, Dr. Ferrier.

November 22, Dr. Aldren Turner, **Muscular Atrophy.**

### A MODERN MEDICAL JOURNAL.

A NEW series of THE HOSPITAL was commenced with the weekly issue dated April 6, 1907. Arrangements were then completed to publish THE HOSPITAL as the modern medical journal, appearing weekly, price 3d., and containing not more than thirty-two pages of literary matter, mainly printed in long primer type, so as to form comfortable reading for the doctor in his carriage.

THE EDITORIAL STAFF.

As its name implies, the new series of THE HOSPITAL will include every section of hospital work. A number of representative members of the medical profession, including some of the most active workers and best writers, now constitute the staff. All sections of medical and surgical work are included in the paper, each section being under the management of an editor selected because of his association with, and knowledge of, the subjects comprised in it.

ITS OBJECTS.

Care will be taken to include in the paper every modern development and all new discoveries, together with the methods of treatment and practice in this country, in the great European schools of medicine, and in the United States. The object will be to endeavour to make each section of the paper both compact and complete; and the whole will be written with the desire to interest, help and keep up to date practitioners engaged in the active service of their profession.

A CLINIC EVERY WEEK.

A Clinical Lecture by some leading member of the medical profession and addressed to graduates is published each week. Thus the earlier numbers have contained clinics on "The Treatment of Acute Pneumonia," by Sir Dyce Duckworth, M.D., LL.D., F.R.C.P.; on "Stiffness of the Spine," by Sir William Bennett, K.C.V.O.; on "Unsoundness of Mind not always Insanity," by G. H. Savage, Esq., M.D., F.R.C.P.; on "The Modern Treatment of Syphilis," by D'Arcy Power, Esq., M.A. M.B., F.R.C.S.; on "Operations on the Bile Passages," by G. H. Makins, Esq., C.B., F.R.C.S.; and on "Common Forms of Dyspepsia in Women," by Robert Saundby, Esq., M.D., F.R.C.P.

WRITERS TO COME.

The following, amongst others, have agreed to contribute Clinics and other articles during the ensuing year:—

**A Clinical Lecture by Wm. Osler, M.D.Oxon., F.R.C.P., F.R.S., LL.D., Regius Professor of Medicine, University of Oxford.**

**A Clinical Lecture by Sir J. Halliday Croom, M.D.Edin., F.R.C.P., F.R.C.S., Consulting Gynæcologist to the Edinburgh Royal Infirmary; Physician Edinburgh Royal Maternity Hospital; Professor of Midwifery at the Edinburgh University.**

**On Syphilitic Cachexia. By Sir Dyce Duckworth, M.D., LL.D., F.R.C.P., Consulting Physician St. Bartholomew's Hospital; Senior Physician Seamen's Hospital, Greenwich.**

**On Waters and Climate. By J. F. Goodhart, M.D., C.M., F.R.C.P., LL.D., Consulting Physician Guy's Hospital.**

**A Clinical Lecture. By Robert Saundby, M.D.Edin., C.M., F.R.C.P., Professor of Medicine Birmingham University; Physician Birmingham General Hospital.**

**A Clinical Lecture. By William Hale White, M.D., F.R.C.S., Physician and Lecturer on Medicine Guy's Hospital.**

**A Clinical Lecture. By H. Gilbert Barling, F.R.C.S.Eng., Professor of Surgery and Ingleby Lecturer University of Birmingham; Surgeon Birmingham General Hospital.**

**On Oral Sepsis in its Relation to Abdominal Disease. By J. H. Dauber, M.N., B.Ch.Oxon., M.A., Gynæcologist Hospital for Women, Soho.**

**On the Importance of Localising Heart Beats. By Arthur Foxwell, M.A., M.D.Cantab., F.R.C.P., Professor of Therapeutics Birmingham University; Physician Queen's Hospital, Birmingham.**

**A Clinical Lecture. By Sir R. Douglas Powell, K.C.V.O., M.D., F.R.C.P., Physician Extraordinary to the King; Consulting Physician, Middlesex and Brompton Hospitals.**

**On Syphilitic Diseases of the Joints. By D'Arcy Powe, M.A., M.B.. F.R.C.S.Eng.; Surgeon and Lecturer on Surgery, St. Bartholomew's Hospital.**

**A Clinical Lecture. By P. H. Pye-Smith, M.D., F.R.C.P., Consulting Physician, Guy's Hospital.**

**A Clinical Lecture. By R. W. Philip, M.A.Edin., M.D., F.R.C.P., Lecturer on Practical Physic, Clinical Medicine, and Diseases of the Chest, Edinburgh School of Medicine; Examiner in Clinical Medicine, University and Royal College of Physicians, Edinburgh; Physician, Edinburgh Royal Infirmary.**

**On The Really Useful in Electro-Therapeutics. By H. Lewis Jones, M.A., M.D., F.R.C.P., Medical Officer in Charge of Electrical Department, St. Bartholomew's Hospital.**

**A Clinical Lecture. By C. R. B. Keetley, F.R.C.S., Senior Surgeon, West London Hospital.**

**A Clinical Lecture. By Jordan Lloyd, M.D., M.S., F.R.C.S., Surgeon, Queen's Hospital, Birmingham; Examiner in Surgery, Durham University.**

**A Clinical Lecture. By Sir George Beatson, K.C.B., M.D., C.M., Examiner in Surgery, Edinburgh University Visiting Surgeon, Glasgow Western Infirmary; Surgeon, Glasgow Cancer Hospital.**

**A Clinical Lecture. By Charters J. Symonds, M.D., M.S., F.R.C.S., Examiner in Surgery, London University; Surgeon and Lecturer on Surgery, Guy's Hospital.**

**On Some Medico-Legal Reminiscences. By A. P. Luff, M.D., F.R.C.P., D.Ph., Lecturer on Medical Jurisprudence and Toxicology, St. Mary's Hospital; Physician, St. Mary' Hospital.**

**On Some Medical Hæmorrhages. By Leonard Williams, M.D., M.R.C.P., Physician, French Hospital.**

**On Certain Urinal Diseases. By Reginald Harrison, F.R.C.S., Consulting Surgeon, St. Peter's Hospital for Stone.**

NEW REMEDIES.

The descriptions of New Remedies will include information as to the directions in which recently introduced drugs can best be used; and the bearing of each new drug upon the treatment of disease from the point of view of its difference from, and advantage over, existing remedies will be discussed. Articles in this section will be based upon actual experience obtained in the treatment of disease.

THE PRACTITIONER'S BOOKSHELF.

New Books will be reviewed promptly and on a new plan. Every endeavour will be made to indicate, of all newly published works, which are best suited to the purposes of the practitioner as sources of reliable and up-to-date information.

A NOVEL DEPARTURE.

There will be a section devoted to the Resident Medical Officer's Department; this will be written from within, under the editorship of a gentleman who is intimately identified with this special work. The interests and welfare of this section of medical men have not heretofore received at the hands of professional journals so much attention as they deserve. The interests of the Resident Medical Officers and of the general practitioner are really one, and it will be the aim of this section of the paper to bring into closer touch these two branches of medical practice.

THE GENERAL PRACTITIONER.

There will be a General Practitioners' Page, and to this contributions are invited from practitioners upon actual experiences in the management of cases and in the treatment and diagnosis of disease.

"The Relaxations of a Medical Man" has proved to be a subject which is helpful and interesting in many ways to many active workers in the profession.

ADMINISTRATION, CONSTRUCTION, AND NURSING.

A special section has been devoted to Hospital Administration, to the planning and construction of hospitals, and to their economical management, together with special articles by matrons and graduate nurses on matters of interest to the busy practitioner.

THE HOSPITAL is successfully providing for the practitioner an up-to-date modern medical journal, which supplies him each week with information and facts of immediate value in the conduct of his practice and in the discharge of the manifold duties which fall to his lot in the course of each day's work.

### GENERAL PRACTITIONERS' CONTRIBUTIONS.

**Important.**

WE propose to devote a special page to General Practitioners' Contributions. We therefore invite from practitioners contributions based upon their experience in the management of cases, and in the treatment and diagnosis of disease; especially shall we be prepared to welcome articles dealing, practically, with treatment, and with the use and value of new remedies and methods.

No article should exceed 1,100 words in length, and, if accepted, one guinea will be paid to the writer after publication. Each communication should be accompanied by a stamped directed envelope for the return of the MS. if found unsuitable.

**The Relaxations of Medical Men.**

We shall also be glad to pay for accepted contributions, from any member of the profession, on the subject of the relaxations of practitioners. This opens up a wide field, as it includes natural history, photography, sport, indoor recreations, and motoring. Whenever possible, original illustrations and photographs should be sent with the MS.

**Suggestions Invited.**

The Editor will welcome suggestions for the establishment of any new section in THE HOSPITAL, and will be glad to supply information on any subject of interest or importance to members of the profession in any part of the world.

THE HOSPITAL *November* 16, 1907.

*Name* ..........................................................

*Address* ..........................................................

..........................................................

This Coupon must accompany manuscript or contributions intended for THE HOSPITAL.

